# Investigation into the Anti-Acne Effects of *Castanea sativa* Mill Leaf and Its Pure Ellagitannin Castalagin in HaCaT Cells Infected with *Cutibacterium acnes*

**DOI:** 10.3390/ijms25094764

**Published:** 2024-04-27

**Authors:** Stefano Piazza, Giulia Martinelli, Nicole Maranta, Carola Pozzoli, Marco Fumagalli, Vincenzo Nicolaci, Elisa Sonzogni, Luca Colombo, Enrico Sangiovanni, Mario Dell’Agli

**Affiliations:** 1 Department of Pharmacological and Biomolecular Sciences “Rodolfo Paoletti”, University of Milan, 20133 Milan, Italy; stefano.piazza@unimi.it (S.P.); giulia.martinelli@unimi.it (G.M.); nicole.maranta@unimi.it (N.M.); carola.pozzoli@unimi.it (C.P.); marco.fumagalli3@unimi.it (M.F.); vincenzo.nicolaci@unimi.it (V.N.); elisa.sonzogni@studenti.unimi.it (E.S.); mario.dellagli@unimi.it (M.D.); 2 Consorzio Castanicoltori di Brinzio, Orino e Castello Cabiaglio, Società Cooperativa Agricola-Varese, 21100 Varese, Italy; info@consorziocastanicoltori.it

**Keywords:** acne, keratinocyte, inflammation, *Castanea sativa* Mill., ellagitannins

## Abstract

Acne vulgaris is a prevalent skin disorder affecting many young individuals, marked by keratinization, inflammation, seborrhea, and colonization by *Cutibacterium acnes* (*C. acnes*). Ellagitannins, known for their antibacterial and anti-inflammatory properties, have not been widely studied for their anti-acne effects. Chestnut (*Castanea sativa* Mill., *C. sativa*), a rich ellagitannin source, including castalagin whose acne-related bioactivity was previously unexplored, was investigated in this study. The research assessed the effect of *C. sativa* leaf extract and castalagin on human keratinocytes (HaCaT) infected with *C. acnes*, finding that both inhibited IL-8 and IL-6 release at concentrations below 25 μg/mL. The action mechanism was linked to NF-κB inhibition, without AP-1 involvement. Furthermore, the extract displayed anti-biofilm properties and reduced CK-10 expression, indicating a potential role in mitigating inflammation, bacterial colonization, and keratosis. Castalagin’s bioactivity mirrored the extract’s effects, notably in IL-8 inhibition, NF-κB inhibition, and biofilm formation at low μM levels. Other polyphenols, such as flavonol glycosides identified via LC-MS, might also contribute to the extract’s biological activities. This study is the first to explore ellagitannins’ potential in treating acne, offering insights for developing chestnut-based anti-acne treatments pending future in vivo studies.

## 1. Introduction

Acne vulgaris is one of the most prevalent skin disorders in young people. It is a chronic disease affecting the pilosebaceous unit within skin areas characterized by a high concentration of sebaceous glands, such as the face and chest [1]. The disease may also persist into adulthood and has a multifactorial etiology, including genetic factors and specific endocrine conditions [2].

At a follicular level, the pathogenesis develops through partially overlapping phases, among which increased keratinization, seborrhea, colonization by *Cutibacterium acnes* (*C. acnes*), and inflammation are fundamental traits [1]. Keratinization is an early event in acne which, coupled with seborrhea, establishes the anoxic environment ideal for *C. acnes* colonization. From the perspective of the bacterium, opportunistic colonization is favored by virulence factors; biofilm formation capacity, rather than proliferation itself, might be a key feature for bacterial outgrowth [3,4]. In analogy with other Gram-positive bacteria, *C. acnes* is recognized by the host through TLR-2 receptors, thus leading to inflammatory signals related to the activation of nuclear factor κB (NF-κB) [5]. Keratinocytes, sebocytes, and resident immune cells contribute to the release of inflammatory mediators in the skin, among which IL-1, TNF-α, IL-6, and IL-8 play a recognized role in the pathogenesis of acne [6].

Due to the complexity of its pathogenesis, multifaceted approaches including the use of natural compounds, affecting multiple targets, are being explored [7]. In this regard, we recently summarized the pre-clinical evidence concerning the anti-inflammatory properties of hydrolyzable tannins, including ellagitannins, at the skin level [8,9]. Other studies have ascribed antimicrobial properties to ellagitannins [10,11]. Moreover, their role in the context of acne is also suggested by their anti-sebum properties and 5α-reductase inhibition [12,13,14,15], although their bioactivity has been scarcely investigated in models of acne infection.

*Castanea sativa* Mill. (*C. sativa*) is a rich source of ellagitannins [16], with it showing anti-inflammatory and anti-*H. pylori* effects in our previous studies [17]. Leaves and other by-products from *C. sativa* are receiving growing attention as valuable sources of polyphenols with potential impact on human health [18,19]. To the best of our knowledge, *C. sativa* extracts and related ellagitannins have never been investigated in models of acne before.

Accordingly, the present work aimed to investigate the bioactivity of a polar extract from a *C. sativa* leaf in a co-culture model of human keratinocytes (HaCaT) and *C. acnes*, thus mimicking inflammatory conditions promoted by the pathogen in human keratinocytes and cell response in terms of the release of pro-inflammatory cytokines and nuclear factor activation.

## 2. Results

### 2.1. Phytochemical Analysis

The leaf of *C. sativa* is a by-product from agricultural processes that is receiving attention as a sustainable source of polyphenols, including hydrolyzable tannins [18,19]. Although chestnut leaves are not typically considered waste, they can be viewed as such following the annual pruning process of the trees. We previously reported that the ellagitannin isomers castalagin and vescalagin, highly present in *C. sativa* bark [16], are bioactive molecules that occur in high amounts in chestnut leaves and in their corresponding hydroalcoholic extracts, which were investigated in this study (about 1% *w/w* of ellagitannin isomers in dry extract) [17]. We measured a high amount of total polyphenols (Folin–Ciocalteu assay), representing more than 25% *w*/*w* of the leaf extract [17].

Other authors have characterized the phytochemical composition of leaf extracts, with them describing flavonol glycosides and hydrolyzable tannins as the main classes of polyphenols [19]. Accordingly, in the framework of this study, we extended our knowledge regarding leaf extract composition by applying qualitative LC-MS analysis; the results reflected those of previous papers and confirmed the presence of quercetin glycosides, kaempferol glycosides (i.e., astragalin), gallotannins (chestanin and chesnatin), and ellagitannins (3,4,5,3′,4′,5′-Hexahydroxydiphenic acid (HHDP), castalagin, and vescalagin) (Table 1).

### 2.2. Bioactivity in HaCaT Cells

It is well established that *C. acnes* triggers the migration of inflammatory cells in the skin through interaction with the TLR-2 receptor, which activates transcription factors, such as NF-κB and AP-1, thus leading to IL-8 and IL-6 release [6]. Since keratinocytes are the first line of interaction with the skin microbiota, we decided to use a co-culture of HaCaT/*C. acnes* to mimic the early events of acneic disease, considering that this approach is the most predictive in studying the interaction of the pathogen with human keratinocytes and the consequent inflammatory cascade [20,21]. For this purpose, as a first step, we measured proinflammatory mediators typically occurring during acneic inflammation, such as IL-8 and IL-6, via ELISA assay. Moreover, the activity of transcription factors involved in their production, namely NF-κB and AP-1, was measured via luciferase assay.

In line with other authors [21], we observed that HaCaT cells release IL-8 after infection with a low bacterial load (O.D. = 0.1) for 24 h (Figure S1A). The release of IL-8 paralleled with IL-6 and the early activation of NF-κB (6 h) (Figure S1B,C); conversely, the activation of AP-1 occurred later, showing a peak after 48 h (Figure S1D). Based on this experimental set, we investigated the anti-inflammatory activity of *C. sativa* leaf extract (0–50 μg/mL) in comparison with the representative ellagitannin castalagin (0–20 μM). As a preliminary step, interference with HaCaT cell viability was excluded using the MTT test after 48 h of treatment, namely the longer time of exposure selected for our experiments (Figure S2).

**Figure 1:**
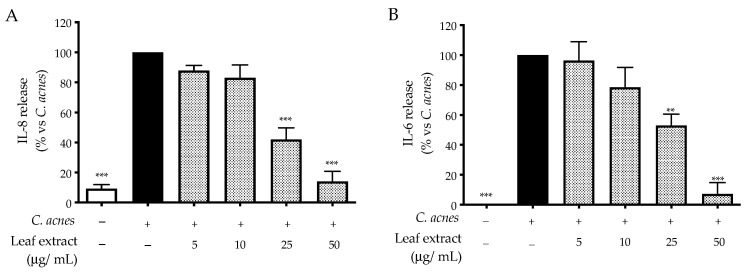
Effect of the leaf extract from *C. sativa* on IL-8 (**A**) and IL-6 (**B**) release, measured via ELISA assay. The HaCaT cells (not stimulated control, white bar) were treated with *C. acnes* O.D. = 0.1 (black bar) and the leaf extract (dots bar) for 24 h. Apigenin (20 μM) was used as a reference inhibitor (−25% of IL-8 and −90% of IL-6 release). Data (*n* = 3) are expressed as the mean (%) ± SEM relative to *C. acnes*, which was arbitrarily assigned the value of 100%. ** *p* < 0.01 and *** *p* < 0.001 vs. *C. acnes*.

The leaf extract inhibited the release of IL-8 and IL-6 induced by *C. acnes* in a concentration-dependent fashion; the IC_50_ values were 18.37 and 22.54 μg/mL, respectively (Figure 1).

**Figure 2:**
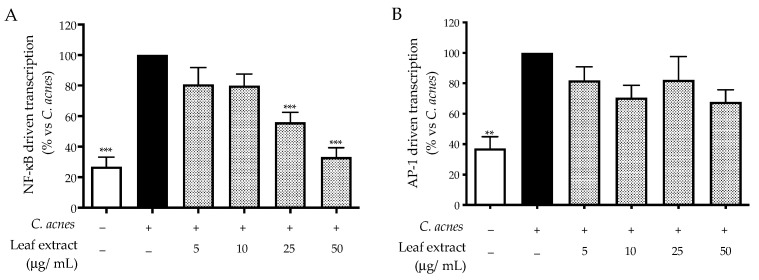
Effect of the leaf extract from *C. sativa* on NF-κB (**A**)- and AP-1 (**B**)-driven transcription, measured via luciferase assay and reporter plasmids. The HaCaT cells (not stimulated control, white bar) were treated with *C. acnes* O.D. = 0.1 (black bar) and the leaf extract (dots bar) for 24 h (**A**) or 48 h (**B**). Apigenin (20 μM) was used as a reference inhibitor of NF-κB activity (−78%). Data (*n* = 3) are expressed as the mean (%) ± SEM relative to *C. acnes*, which was arbitrarily assigned the value of 100%. ** *p* < 0.01 and *** *p* < 0.001 vs. *C. acnes*.

Consequently, we selected NF-κB and AP-1 as potential targets able to explain the mechanism of action. The leaf extract impaired NF-κB-driven transcription with an IC_50_ equal to 16.13 μg/mL (Figure 2A), while that of AP-1 was only slightly reduced at the highest concentration tested (50 μg/mL) (Figure 2B), and the effect was not statistically relevant. Of note, our data reflected the experimental settings concerning stimulation with *C. acnes*, which suggested NF-κB as the main regulator of pro-inflammatory interleukins.

**Figure 3:**
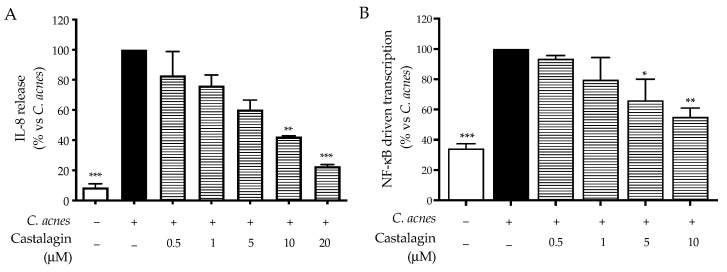
Effect of castalagin on IL-8 release (**A**) and NF-κB-driven transcription (**B**), measured via ELISA and luciferase assay, respectively. The HaCaT cells (not stimulated control, white bar) were treated with *C. acnes* O.D. = 0.1 (black bar) and castalagin (horizontal lines bar) for 24 h. Apigenin (20 μM) was used as a reference inhibitor (−20% of IL-8 release and −77% of NF-κB activity). Data (*n* = 3) are expressed as the mean (%) ± SEM relative to C. *acnes*, which was arbitrarily assigned the value of 100%. * *p* < 0.05, ** *p* < 0.01, and *** *p* < 0.001 vs. *C. acnes*.

Our previous work, considering *H. pylori*-induced inflammation in gastric cells, clearly correlated the bioactivity of *C. sativa* leaf extract with castalagin content [17]; thus, ellagitannin was also investigated in the co-culture mimicking the acneic disease, as a representative compound. For this purpose, we selected IL-8 release and NF-κB-driven transcription as relevant outcomes. Once again, the inhibitory activity of castalagin was observed for both inflammatory markers, thus confirming the parallelism between ellagitannin and the leaf extract (Figure 3A,B).

The values of IC_50_ on inflammatory markers induced by *C. acnes* are summarized and compared in Table 2.

At this point in our investigation, we wondered whether the leaf extract and castalagin might interfere with further inflammatory pathways involved in acne and converging in NF-κB signaling. It is common knowledge that the TLR and IL-1R pathways activate NF-κB through an enzymatic cascade depending on MyD88 [22]. IL-1R can be activated by IL-1α and IL-1β within the inflamed comedones of acne patients [6].

**Figure 4:**
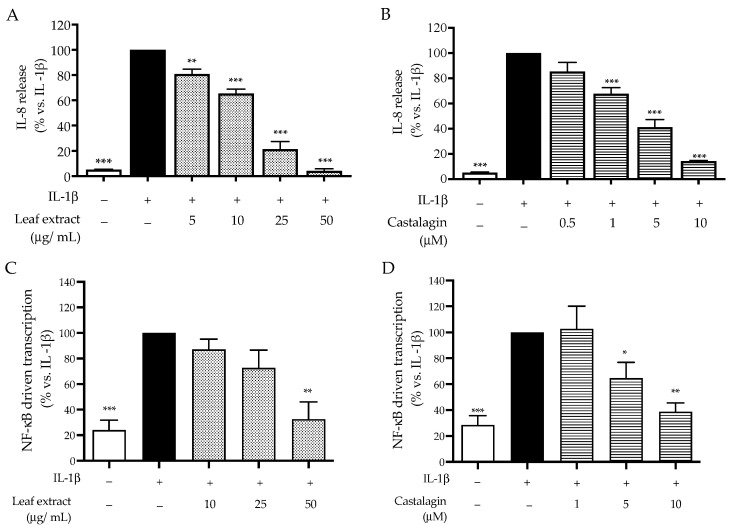
Effect of the leaf extract from *C. sativa* and castalagin on IL-8 release (**A**,**B**) and NF-κB-driven transcription (**C**,**D**), measured via ELISA and luciferase assay, respectively. The HaCaT cells (not stimulated control, white bar) were treated with IL-1β 10 ng/mL (black bar) and the leaf extract (dots bar) or castalagin (horizontal lines bar) for 6 h. Data (*n* = 3) are expressed as the mean (%) ± SEM relative to IL-1β, which was arbitrarily assigned the value of 100%. * *p* < 0.05, **p < 0.01, and *** *p* < 0.001 vs. *C. acnes*.

With this in mind, we decided to further investigate the bioactivity of the leaf extract and castalagin against IL-1β stimulation in HaCaT cells. As expected, the consequent release of IL-8 was reduced following treatment at concentrations comparable to the previous data (Figure 4A,B). Accordingly, the activation of NF-κB was also impaired, thus confirming the hypothesis regarding the anti-inflammatory mechanism (Figure 4C,D). The values of IC_50_ regarding the effect on inflammatory markers induced by IL-1β are summarized and compared in Table 3.

Keratinocytes play a relevant role in the early inflammation of acne as well as in the formation of comedones through keratinization. The molecular events that link *C. acnes* and keratinization are still unclear, but it is known that signals from bacteria, hormones, and inflammatory mediators are involved [1,23].

**Figure 5:**
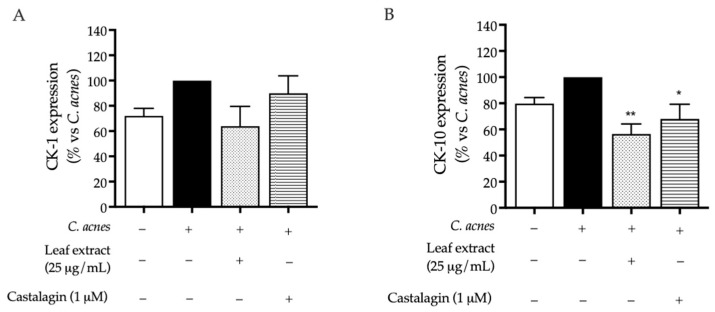
Effect of the leaf extract from *C. sativa* and castalagin on CK-1 (**A**) and CK-10 (**B**) expression, measured via ELISA. The HaCaT cells (not stimulated control, white bar) were treated with *C. acnes* O.D. = 0.1 (black bar) and the leaf extract (dots bar) or castalagin (horizontal lines bar) for 48 h. Data (*n* = 3) are expressed as the mean (%) ± SEM relative to C. *acnes*, which was arbitrarily assigned the value of 100%. * *p* < 0.05 and ** *p* < 0.01 vs. *C. acnes*.

**Figure 6:**
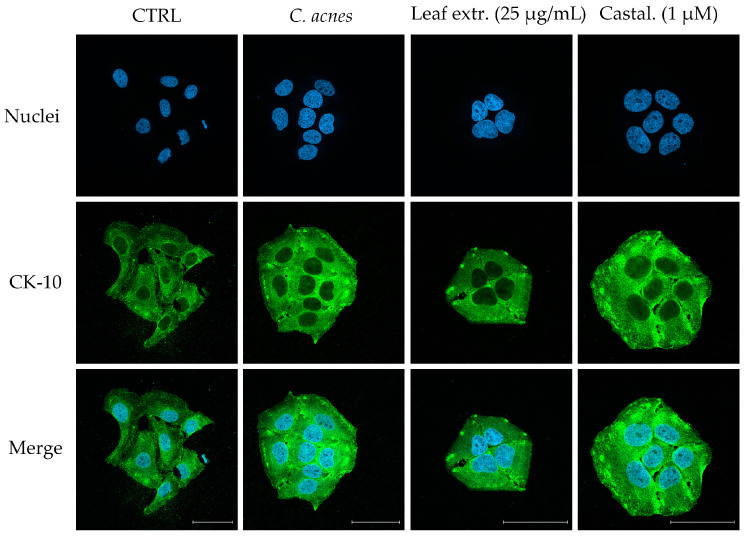
Effect of the leaf extract from *C. sativa* and castalagin on CK-10 expression, measured using confocal microscopy (63× objective; 50 μm scale). The HaCaT cells were treated with *C. acnes* O.D. = 0.1 and the leaf extract or castalagin for 48 h. The localization of nuclei and CK-10 was detected using blue (DAPI) and green (anti-mouse AlexaFluor^®^ 488) fluorescent probes, respectively.

To explore the potential impact of *C. sativa* leaf extract on keratinization, we measured the expression of CK-1 and CK-10, cytokeratins expressed in the superbasal layers of the epidermis, after 48 h of co-culture. To exclude the effect of *C. acnes* relying on cell proliferation, cytotoxicity was analyzed using trypan blue count and the MTT test, respectively. In our experimental conditions, *C. acnes* caused a slight but not significant increase in both cytokeratins, while the leaf extract (25 μg/mL) and, in a minor part, castalagin (1 μM) showed an opposite effect (Figure 5A,B). Since it was significant for CK-10 only, the expression of CK-10 was investigated through an additional technique to support the previous results. The immunofluorescence images shown in Figure 6 (Figure 6) depict similar data, thus suggesting that the leaf extract might counteract abnormal differentiation; on the contrary, castalagin did not show fluorescence inhibition, suggesting a marginal role in CK-10 regulation and suggesting that other components of the extract might be responsible for this biological activity.

### 2.3. Antibacterial Activity against C. acnes

The colonization of the pilosebaceous unit by *C. acnes* is a crucial event in acne [3,4]. According to previous articles, ellagitannins exhibit antibacterial properties against Gram-negative and Gram-positive bacteria [10,11]. Thus, we tested the antibacterial effect of the leaf extract and pure castalagin against *C. acnes*, either by means of inhibition of bacterial growth (MIC: minimum inhibitory concentration) or biofilm formation. The first was measured using the microbroth dilution method, while the second was measured via crystal violet assay. Based on our previous experiments demonstrating antibacterial activity against *H. pylori* at the concentration of 25 μM [17], castalagin was also tested and its activity was compared with that of the extract.

**Figure 7:**
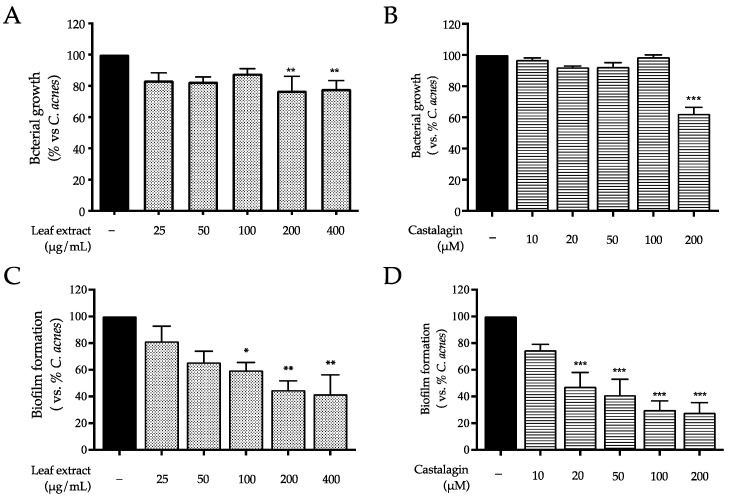
Effect of the leaf extract from *C. sativa* and castalagin on bacterial growth (**A**,**B**) and biofilm formation (**C**,**D**), measured using the broth microdilution test and crystal violet assay. *C. acnes* (black bar) was treated with the leaf extract (dots bar) or castalagin (horizontal lines bar) for 48 h. Data (*n* = 3) are expressed as the mean (%) ± SEM relative to *C. acnes*, which was arbitrarily assigned the value of 100%. * *p* < 0.05, ** *p* < 0.01, and *** *p* < 0.001 vs. *C. acnes*.

Surprisingly, the leaf extract showed a negligible effect (−20%) on the growth of *C. acnes* at the concentration of 200 and 400 μg/mL (Figure 7A), while castalagin demonstrated only a slight inhibitory effect at the highest concentration tested of 200 μM (Figure 7B). Unexpectedly, when performing the crystal violet assay to stain the bacterial biofilm, a stronger effect was observed. In fact, the leaf extract significantly impaired biofilm formation within the concentration range of 100–400 μg/mL; once again, castalagin showed the same bioactivity starting at 20 μM (Figure 7C,D).

Antibiotic therapy might perturbate the skin microbiota and induce antibiotic resistance in acne patients [24]; thus, we decided to investigate the antibacterial properties of co-treating the leaf extract with the gold-standard antibiotic erythromycin. Through this experiment, we aimed to address a potential synergistic effect, which might sustain a combination treatment to reduce the dose of antibiotics.

**Figure 8:**
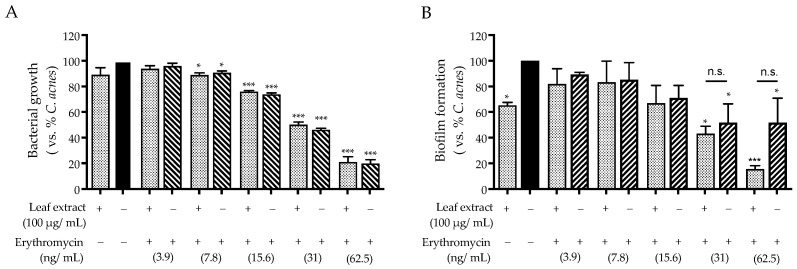
Effect of the combination of the leaf extract from *C. sativa* (100 μg/mL) with erythromycin on bacterial growth (**A**) and biofilm formation (**B**), measured using the broth microdilution test and crystal violet assay. *C. acnes* O.D. = 0.1 (black bar) was treated with erythromycin alone (oblique line bar) or combined with castalagin (dots bar) for 48 h. Data (*n* = 3) are expressed as the mean (%) ± SEM relative to *C. acnes*, which was arbitrarily assigned the value of 100%. * *p* < 0.05 and *** *p* < 0.001 vs. *C. acnes*.

For this purpose, we first measured the concentration range in which erythromycin exhibited its antibiotic effect (0–62.5 ng/mL). Then, a selected concentration of leaf extract (100 μg/mL) was added to each concentration of erythromycin tested. As expected from previous data, the antibiotic MIC was not altered by the addition of the leaf extract (Figure 8A). On the contrary, the effect of erythromycin on biofilm formation was evident at lower concentrations when combined with the leaf extract (Figure 8B). Unfortunately, the observed difference was not significant.

## 3. Discussion

Acne vulgaris is a multifactorial disorder of the pilosebaceous unit. At the present state of the art, *C. acnes* infection and skin inflammation represent the major targets of pharmacological and cosmetic strategies. The therapeutic approach depends on the severity of the disease and aims to reduce the inflammatory state and prevent the formation of fibrotic scars with a long-term impact on both esthetic and psychological health [24,25]. However, the current approach is also limited by several challenges. The use of antibiotics in patients with severe acne is related to increasing antibiotic resistance and the perturbation of skin ecology [24]. The use of retinoids is limited by teratogenic effects and relatively frequent side effects, such as xerosis, thus reducing adherence to therapy [25]. Combination therapies, including natural compounds showing anti-inflammatory activity with a mild impact on skin microbiota, might be useful for the management of acne [7].

Ellagitannins have been investigated for their anti-inflammatory, antibacterial, and prebiotic effects [9,10,11,26], but their potential role in acne is poorly investigated.

Leaves from *C. sativa* are by-products of agricultural processes that are receiving great attention as sustainable sources of polyphenols, including ellagitannins [18,19]. We previously reported that hydroalcoholic extract from *C. sativa* leaf contains a significant amount of ellagitannin including the isomers castalagin and vescalagin (about 1% *w*/*w*), showing anti-inflammatory and antibacterial effects against *H. pylori* [17]. Herein, we extended the characterization of the same extract via qualitative LC-MS (Table 1), which revealed the presence of organic acids, flavonol glycosides, and hydrolyzable tannins, in line with other authors [19].

The present investigation also compared the bioactivity of *C. sativa* leaf extract and castalagin in a co-culture model of human keratinocytes (HaCaT) and *C. acnes*. Our outcomes were selected by referring to the pathogenesis of acne, with a particular focus on the role of keratinocytes. For this purpose, markers of inflammation and differentiation were investigated; moreover, antibacterial and anti-biofilm investigations were performed.

The leaf extract inhibited the release of IL-8 and IL-6, with IC_50_ values below 25 μg/mL. The mechanism of action was mainly attributed to the impairment of NF-κB, which was demonstrated at the same concentrations. Of note, castalagin reflected the anti-inflammatory activity of the leaf extract since IL-8 release and NF-κB were counteracted at concentrations lower than 5 μM.

Our data concerning the mechanism of action were supported by further experiments. The same inhibitory activity was observed during the stimulation of HaCaT cells with IL-1β (Table 3), an interleukin known to induce inflammation through pathways shared across the TLR-2 receptor.

Although castalagin reflected the bioactivity of the leaf extract in qualitative terms, its IC_50_ values were not sufficient to completely explain those of the leaf extract. Considering that castalagin and its isomer account for about 1% *w*/*w* of the extract, the IC_50_ value of ellagitannins would have been lower. This suggestion also emerged from our previous study [17], in which we supposed that other hydrolyzable tannins with a similar structure to castalagin might participate in the bioactivity of the whole extract. Moreover, other polyphenols present in the extract, such as flavonols and ellagic acid, have already been cited for their anti-inflammatory effects against acne [27].

The molecular events behind the process of keratinization occurring in acne are still unclear. Factors involved in the alteration of skin turnover include bacterial products (propionic acid) [28], cytokines, and hormones [23].

Through co-culture studies, it has been demonstrated that *C. acnes* induces markers of terminal differentiation in human keratinocytes [29,30]. Of note, several authors, including our group, have demonstrated that *C. acnes* increases the activity of AP-1 [21], which plays an important role in the differentiation of keratinocytes [31].

Despite ellagitannins being implicated in skin barrier protection, their specific role in acne is poorly investigated [13]. While proper evaluations ideally include primary keratinocytes and 3D cultures, the main objective of this study was to conduct a preliminary assessment of the potential role of leaf extract and castalagin in differentiation. Our data suggest that the leaf extract may counteract the acceleration of turnover caused by *C. acnes* in our settings, by means of CK-1 and CK-10 expression (Figure 5 and Figure 6). The role of castalagin in this effect is unclear, thus suggesting the involvement of other polyphenols. However, further experiments should be devoted to the investigation of this specific aspect.

Regarding *C. acnes* as a direct target, our experiments demonstrated an anti-biofilm rather than an antimicrobial effect (Figure 7). Of note, the activity of the leaf extract (100 μg/mL) might cause an additional effect when used in co-treatment with erythromycin (Figure 8). For this purpose, it is noteworthy that *C. acnes* not only plays an opportunistic role in disease but also exerts positive effects on healthy skin homeostasis [3]. The anti-biofilm effect might be considered a strategy for the maintenance of skin ecology during novel anti-acne treatments. For these reasons, *C. sativa* leaf extract could be a valuable adjunct in acne management therapies.

In conclusion, to the best of our knowledge, this is the first study aimed at validating the potential role of *C. sativa* leaf in acneic disease. As a valuable and sustainable source of ellagitannins, we selected the leaf of *C. sativa*, characterized by the presence of castalagin. Our data, obtained in a co-culture model of keratinocytes, might be informative for the validation of its anti-inflammatory and anti-biofilm properties through future in vivo studies. Of note, bioactivity was observed at concentrations easily achievable after topical application of milligrams of extract. Accordingly, formulation strategies should also be developed to deliver the extract not only to the epidermis but also to the pilosebaceous unit.

Future studies should consider the effect of *C. sativa* extract and pure castalagin in an in vivo model of acne pathology. However, it will be important to assess the bioavailability of active molecules within the pilosebaceous unit to ensure the efficacy of *C. sativa* leaf extract in practical therapeutic applications.

## 4. Materials and Methods

### 4.1. Plant Material and Extraction

The leaves of *C. sativa* (var. venegon) were collected during the pruning process and extracted as previously reported [17]. In brief, plant material was obtained through a consortium of farmers located in the regional natural park “Campo dei Fiori” (Castello Cabiaglio and Brinzio areas, Varese, Italy).

Dried leaves (2.5 g) were milled and extracted twice with 50 mL of ethanol/water 50:50 (hydroalcoholic extract) for 4 and 16 h, respectively, at room temperature under dark conditions. The plant residue was removed using Supervelox filter paper, while the filtrate was concentrated by rotavapor for 2 h at 40 °C and then frozen at −80 °C overnight. Finally, a freeze-dried extract was produced and maintained at −20 °C. The extracts were dissolved in sterilized distilled water and DMSO (H_2_O:DMSO 50:50, 25 mg/mL) before being stored in aliquots at −20 °C until the biological experiments.

### 4.2. Cell Culture

Human immortalized keratinocytes (HaCaT cells, passage from 30 to 50; CVCL-0038; Cell Line Service, Eppelheim, Germany) were cultured using high-glucose DMEM (Merck Life Science, Milano, Italy), supplemented with FBS 10%, penicillin/streptomycin 1% (Pen Strep Gibco^TM^; Thermo Fisher Scientific, Monza, Italy), and glutamine 1% (2 mM) (Thermo Fisher Scientific, Monza, Italy). For the subculture, the cells were detached after calcium chelation (EDTA 0.25%) using Trypsin-EDTA 0.25% mixture (Gibco^TM^; Thermo Fisher Scientific, Monza, Italy). Then, 1.5 × 10^5^ cells were seeded in a 75 cm^2^ flask (Primo^®^; Euroclone, Pero, Italy), where they proliferated upon confluency after 48–72 h, under the controlled atmosphere of the incubator (37 °C, 5% CO_2_, 90% humidity).

*Cutibacterium acnes* (*C. acnes*) 6919^TM^ was purchased from ATCC^®^ cell bank (LGC standards s.r.l., Milano, Italy), with it being reported that the first colony was sampled from facial acne. The bacterium was cultivated in solid-agar blood Petri dishes, containing agar 1% (Merck Life Science, Milan, Italy), defibrinated sheep blood 5%, and RCM medium (TCS Biosciences Ltd., Oxoid, Hampshire, UK). For this purpose, the Petri dishes were inoculated with 100 μL of bacterial stock and kept under an anaerobic atmosphere (GasPak^TM^ EZ CO_2_ generator, Becton Dickinson, Sparks, MD, USA) at 37 °C for 48–72 h in a cell incubator. Aliquots of bacteria (O.D.600 nm = 5) were stored in cryovials at −80 °C inside a medium suitable for cryopreservation, composed of RCM 30%, glycerol 20%, and defibrinated sheep blood 50%.

### 4.3. Treatment of HaCaT Cells and Co-Culture with C. acnes

The HaCaT cells were seeded in 24-well plates or 96-well plates (Falcon^®^; Corning Life Sciences, Amsterdam, The Netherlands) and grown with supplemented DMEM before treatment. Stimulation was performed with IL-1β (10 ng/mL) or *C. acnes* co-culture.

In the case of IL-1β stimulation, concomitant treatment with the leaf extract or castalagin, diluted in serum-free DMEM, was carried out for 6 h.

For the co-culture experiments, *C. acnes* was collected from the Petri dishes, suspended with 1 mL of PBS 1×, counted using optical absorbance at 600 nm (Biochrom Libra S22 UV/VIS spectrophotometer, Waterbeach Cambridge, UK), and finally diluted in 24-well plates to reach a standardized load (O.D. 600 nm = 0.1), thus obtaining the co-culture with HaCaT cells. The concomitant treatment with *C. sativa* leaf extract or castalagin, diluted in antibiotic- and serum-free DMEM, ranged from 24 h to 48 h depending on the biological outcome. Apigenin 20 μM (PhytoLab GmbH & Co. KG, Vestenbergsgreuth, Germany) was used as a reference anti-inflammatory polyphenol, as previously reported [20].

### 4.4. Cell Viability

HaCaT cell morphology was observed via light microscope inspection before and after each treatment. Cell viability was measured at the end of treatment (6–48 h) through the use of the 3,4,5-dimethylthiazol-2-yl-2-5-diphenyl tetrazolium bromide (MTT) test. No cytotoxicity was observed after treatment with the leaf extract (0–50 μg/mL) or castalagin (0–20 μM, M. W. = 934 g/mol) for the longer period of treatment, corresponding to 48 h (Figure S2).

### 4.5. Immunoassays

#### 4.5.1. ELISA Assay

The HaCaT cells were seeded in 24-well (6 × 10^4^ cells) plates for 48 h; then, the co-culture with *C. acnes* was obtained as previously described and treated with the leaf extract or castalagin for 24 h. Human IL-8 and IL-6 were measured in the co-culture medium at the end of each experiment using sandwich ELISA kits (PeproTech, London, UK), according to the manufacturer’s instructions [20]. In brief, EIA/RIA 96-well plates (Merck Life Science, Milan, Italy) were prepared using an anti-human IL-8/IL-6 coating antibody. The day after, BSA 1%/PBS 1× solution was added for 1 h to block unspecific sites of binding; then, 100 μL of the sample was added for 2 h. Finally, a biotinylated anti-human IL-8/IL-6 was used to complete the sandwich construct. The amount of proteins was measured via the acquisition of the absorbance at 405 nm (Victor X3; PerkinElmer, Milan, Italy), after adding the HRP-biotin construct needed to mediate the quantitative oxidation of the ABTS substrate (Merck Life Science, Milan, Italy). The absorbance of the sample was compared with a calibration curve made by standard proteins ranging from 0 to 1500 pg/mL.

Similarly, human cytokeratins (CK-1 and CK-10) were investigated after treating the co-culture with the leaf extract or castalagin for 48 h. For this purpose, the HaCaT cells were grown for only 24 h in 96-well plates (2 × 10^4^ cells) to obtain cells with a low grade of confluency and avoid differentiation to corneocyte-like cells [20,32]; then, cytokeratins were detected via immunoassay on adherent cells. Firstly, the cells were washed (PBS 1×) and fixed using PBS 1×/PFA 4% solution for 15 min. Then, the cells were washed again and blocked-permeabilized using PBS 1×/BSA 5%/Triton-X 0.3% solution for 1 h. Finally, mouse anti-human CK-1 (LHK1, NB100-2756) or CK-10 antibody (3C2F5, NBP2-61736) (Novus biologicals, Milan, Italy) at the final concentration of 0.5 μg/mL (100 μL/well) was added to cells for 2 h. Again, the rate of expression was measured as colorimetric conversion of ABTS (405 nm) by the HRP conjugated IgG anti-mouse antibody (Merck Life Science, Milan, Italy), which was added at the final concentration of 0.25 μg/mL for 30 min.

#### 4.5.2. Confocal Microscopy Analysis

The HaCaT cells were cultured on glass coverslips in 24-well plates (2 × 10^4^/well) for 24 h. Then, co-culture with *C. acnes* and the treatment with the leaf extract or castalagin were conducted as previously described, for 48 h. At the end of treatment, the cells were fixed using PBS 1×/PFA 4% solution for 15 min, and then, they were washed with PBS 1X. The blocking–permeabilizing solution was added (BSA 5%, Triton-X 0.3%, in PBS 1X) for 1 h, and then, immunostaining with mouse anti-human CK10 (3C2F5, NBP2-61736) (1 μg/mL) (Novus biologicals, Milan, Italy) was performed as previously described [20]. After overnight incubation at 4 °C, the AlexaFluor^®^ 488-conjugated anti-mouse antibody #150113 (Abcam, Cambridge, CB2 0AX, UK) was added for 1 h, and then, the cells were washed three times and mounted on glass slides with ProLong Gold Antifade DAPI (Cell Signaling, MA, USA). The fluorescent images were acquired through the use of confocal microscopy (LSM 900, Zeiss, Oberkochen, Germany).

### 4.6. NF-κB- and AP-1-Driven Transcription

The transcriptional activity of NF-κB and AP-1 was investigated via luciferase assay, as previously reported [20]. In brief, the HaCaT cells were cultured on glass coverslips in 24-well plates (6 × 10^4^/well) for 48 h; then, the cells were transfected with NF-κB-Luc or AP-1-Luc reporter plasmids (250 ng/well, for both), containing the luciferase gene under the control of κB or AP-1 responsive elements, respectively. NF-κB-Luc was a gift from Dr. N. Marx (Department of Internal Medicine-Cardiology, University of Ulm; Ulm, Germany), while AP-1-Luc was obtained from the Addgene non-profit repository (Addgene, LGC Standards, Teddington, UK). Transfection was conducted using Lipofectamine^®^ 3000 Transfection Reagent (Invitrogen^®^; Thermo Fisher Scientific, Monza, Italy) following the manufacturer’s instructions. The day after, the cells were stimulated with IL-1β or *C. acnes* co-culture, as described. The concomitant treatment with the leaf extract or castalagin was conducted for 6 h or 24 h, respectively.

### 4.7. Microbiological Assays

The activity of the leaf extract and castalagin against the growth of *C. acnes* was evaluated through the use of the serial dilution method. Briefly, the tested substances were diluted in RCM and placed in a 96-U-bottom well plate (Greiner Bio-one, Milan, Italy) with a final volume of 100 μL. Then, 100 μL of RCM containing the bacterial suspension (final O.D. = 0.1) was added to the samples. The plate was incubated for 24 h under anaerobic conditions to allow for the proliferation of *C. acnes*; then, the optical density at 600 nm was read (Victor X3; PerkinElmer, Milan, Italy).

The formation of biofilm was measured following the same procedure of treatment, after which planktonic cells were removed and adherent cells were stained with crystal violet (Remel Europe Ltd., Dartford, United Kingdom) 0.1% in distilled water. Then, the excess was removed by washing the cells three times with distilled water. Stained cells were extracted with 33% acetic acid, and the absorbance of the resulting solution was measured at 600 nm (Victor X3; PerkinElmer, Milan, Italy). The antibiotic erythromycin (Merck Life Science, Milan, Italy) was used as a reference inhibitor.

### 4.8. LC-MS

Qualitative analysis of the extracts was performed using liquid chromatography (system Surveyor MS PUMP PLUS, Thermo Fisher Scientific, Monza, Italy) coupled with mass spectrometry (LTQ ion-trap mass spectrometer, Thermo Fisher Scientific, Monza, Italy) equipped with an ESI (-) source.

The compounds were separated with a Synergi 4 μm Hydro-RP 80° A LC Column 150 × 4.6 mm (Phenomenex, Torrance, CA, USA) using a mobile phase consisting of water with 0.1% formic acid (A) and acetonitrile (B) set as follows: 0–1 min (5% B), 1–10 min (5–100% B), 10–15 min (100% B), and 15–25 min (100–5% B).

The extracts were injected at 0.1 mg/mL in a volume of 10 μL. The results were analyzed with Xcalibur™ (Thermo Fisher Scientific, Monza, Italy). Putative identification of individual compounds was conducted by comparing *m*/*z* and MS/MS with the literature data [33,34,35].

### 4.9. Statistical Analysis

All data were expressed as the mean ± SEM of at least three independent experiments. ELISA assays were analyzed through the use of unpaired one-way analysis of variance (ANOVA), followed by the Holm–Šídák post hoc test. Statistical analyses were performed using GraphPad Prism 9.0 software (GraphPad Software Inc., San Diego, CA, USA). Values of *p* < 0.05 (*) were considered statistically significant.

## Figures and Tables

**Table 1:** LC-MS analysis of hydroalcoholic extract from a *Castanea sativa* Mill. leaf.

Tentative Identification	R.T.	*m*/*z* [M-H]^−^	MS^2^ Fragments
HHDP acid	5.31	337	293, 169.07
Castalagin/vescalagin	6.14	933	914.92, 897.21, 631.15, 612.90, 301.11, 924.05
Quinic acid	6.48	191	144.95, 129.03, 147.00, 152.86, 172.91
Chesnatin	7.39	637	593.23, 469.18, 467.07
Cretanin	7.79	469	169.04, 305.12, 303.88, 261.14, 306.02, 262.17, 243.22, 393.43
Chestanin	7.86	937	467.11, 469.07, 637.07
Quercetin glucuronide	9.05	477	315.08, 301.09
Quercetin glucoside	9.16	463	301.03, 300.03, 343.20, 151.05
Ellagic acid	9.61	301	301.00, 257.10, 185.02, 232.90, 229.07, 284.02
Astragalin	9.67	447	314.97, 316.07, 284.08, 285.17, 379.31

R.T., retention time; HHDP acid: 3,4,5,3′,4′,5′-Hexahydroxydiphenic acid.

**Table 2:** Summary of the IC_50_ of castalagin and *C. sativa* leaf extract measured in a co-culture of HaCaT cells with *C. acnes*.

	Inflammatory Markers: IC_50_ Mean (CI 95%)	
	IL-8 Release	NF-κB Activity
Leaf extract (μg/mL)	18.37 (14.52 to 24.09)	16.12 (10.13 to 25.75)
Castalagin (μM)	3.66 (1.95 to 6.87)	4.06 (1.46 to 11.29)

IC_50_, 50% inhibitory concentration; CI 95%, 95% confidence interval.

**Table 3:** Summary of the IC_50_ of castalagin and the *C. sativa* leaf extract measured in HaCaT stimulated by IL-1β.

	Inflammatory Markers: IC_50_ Mean (CI 95%)	
	IL-8 Release	NF-κB Activity
Leaf extract (μg/mL)	11.97 (10.13 to 14.14)	34.71 (21.86 to 55.10)
Castalagin (μM)	2.24 (1.55 to 3.24)	5.49 (3.38 to 8.90)

IC_50_, 50% inhibitory concentration; CI 95%, 95% confidence interval.

## Data Availability

The data that support the findings of this study are available from the corresponding author upon request.

## Supplementary Materials

The following supporting information can be downloaded at: https://www.mdpi.com/article/10.3390/ijms25094764/s1.

## References

[1] Williams H.C., Dellavalle R.P., Garner S. (2012). Acne vulgaris. Lancet.

[2] Bhate K., Williams H.C. (2013). Epidemiology of acne vulgaris. Br. J. Dermatol..

[3] Mayslich C., Grange P.A., Dupin N. (2021). Cutibacterium acnes as an Opportunistic Pathogen: An Update of Its Virulence-Associated Factors. Microorganisms.

[4] Dreno B., Pecastaings S., Corvec S., Veraldi S., Khammari A., Roques C. (2018). Cutibacterium acnes (Propionibacterium acnes) and acne vulgaris: A brief look at the latest updates. J. Eur. Acad. Dermatol. Venereol..

[5] Kim J. (2005). Review of the innate immune response in acne vulgaris: Activation of Toll-like receptor 2 in acne triggers inflammatory cytokine responses. Dermatology.

[6] Firlej E., Kowalska W., Szymaszek K., Rolinski J., Bartosinska J. (2022). The Role of Skin Immune System in Acne. J. Clin. Med..

[7] Trivedi M.K., Bosanac S.S., Sivamani R.K., Larsen L.N. (2018). Emerging Therapies for Acne Vulgaris. Am. J. Clin. Dermatol..

[8] Piazza S., Fumagalli M., Khalilpour S., Martinelli G., Magnavacca A., Dell’Agli M., Sangiovanni E. (2020). A Review of the Potential Benefits of Plants Producing Berries in Skin Disorders. Antioxidants.

[9] Piazza S., Fumagalli M., Martinelli G., Pozzoli C., Maranta N., Angarano M., Sangiovanni E., Dell’Agli M. (2022). Hydrolyzable Tannins in the Management of Th1, Th2 and Th17 Inflammatory-Related Diseases. Molecules.

[10] Funatogawa K., Hayashi S., Shimomura H., Yoshida T., Hatano T., Ito H., Hirai Y. (2004). Antibacterial activity of hydrolyzable tannins derived from medicinal plants against Helicobacter pylori. Microbiol. Immunol..

[11] Buzzini P., Arapitsas P., Goretti M., Branda E., Turchetti B., Pinelli P., Ieri F., Romani A. (2008). Antimicrobial and antiviral activity of hydrolysable tannins. Mini Rev. Med. Chem..

[12] Koseki J., Matsumoto T., Matsubara Y., Tsuchiya K., Mizuhara Y., Sekiguchi K., Nishimura H., Watanabe J., Kaneko A., Hattori T. (2015). Inhibition of Rat 5alpha-Reductase Activity and Testosterone-Induced Sebum Synthesis in Hamster Sebocytes by an Extract of Quercus acutissima Cortex. Evid. Based Complement. Altern. Med..

[13] Yin J., Hwang I.H., Lee M.W. (2019). Anti-acne vulgaris effect including skin barrier improvement and 5alpha-reductase inhibition by tellimagrandin I from Carpinus tschonoskii. BMC Complement. Altern. Med..

[14] Kim M., Yin J., Hwang I.H., Park D.H., Lee E.K., Kim M.J., Lee M.W. (2020). Anti-Acne Vulgaris Effects of Pedunculagin from the Leaves of Quercus mongolica by Anti-Inflammatory Activity and 5alpha-Reductase Inhibition. Molecules.

[15] You J., Ji H., Roh K.-B., Cho E., Chajra H., Frechet M., Park D., Jung E. (2022). Anti-acne effects of Castanea crenata bur extract and identification of active compound. Appl. Biol. Chem..

[16] Comandini P., Lerma-Garcia M.J., Simo-Alfonso E.F., Toschi T.G. (2014). Tannin analysis of chestnut bark samples (Castanea sativa Mill.) by HPLC-DAD-MS. Food Chem..

[17] Piazza S., Martinelli G., Fumagalli M., Pozzoli C., Maranta N., Giavarini F., Colombo L., Nicotra G., Vicentini S.F., Genova F. (2023). Ellagitannins from Castanea sativa Mill. Leaf Extracts Impair *H. pylori* Viability and Infection-Induced Inflammation in Human Gastric Epithelial Cells. Nutrients.

[18] Braga N., Rodrigues F., Oliveira M.B. (2015). Castanea sativa by-products: A review on added value and sustainable application. Nat. Prod. Res..

[19] Formato M., Vastolo A., Piccolella S., Calabro S., Cutrignelli M.I., Zidorn C., Pacifico S. (2022). Castanea sativa Mill. Leaf: UHPLC-HR MS/MS Analysis and Effects on In Vitro Rumen Fermentation and Methanogenesis. Molecules.

[20] Piazza S., Martinelli G., Vrhovsek U., Masuero D., Fumagalli M., Magnavacca A., Pozzoli C., Canilli L., Terno M., Angarano M. (2022). Anti-Inflammatory and Anti-Acne Effects of Hamamelis virginiana Bark in Human Keratinocytes. Antioxidants.

[21] Grange P.A., Raingeaud J., Calvez V., Dupin N. (2009). Nicotinamide inhibits Propionibacterium acnes-induced IL-8 production in keratinocytes through the NF-kappaB and MAPK pathways. J. Dermatol. Sci..

[22] Jain A., Kaczanowska S., Davila E. (2014). IL-1 Receptor-Associated Kinase Signaling and Its Role in Inflammation, Cancer Progression, and Therapy Resistance. Front. Immunol..

[23] Briganti S., Flori E., Mastrofrancesco A., Ottaviani M. (2020). Acne as an altered dermato-endocrine response problem. Exp. Dermatol..

[24] Dessinioti C., Katsambas A. (2022). Antibiotics and Antimicrobial Resistance in Acne: Epidemiological Trends and Clinical Practice Considerations. Yale J. Biol. Med..

[25] Zaenglein A.L., Pathy A.L., Schlosser B.J., Alikhan A., Baldwin H.E., Berson D.S., Bowe W.P., Graber E.M., Harper J.C., Kang S. (2016). Guidelines of care for the management of acne vulgaris. J. Am. Acad. Dermatol..

[26] Yin Y., Martinez R., Zhang W., Estevez M. (2023). Crosstalk between dietary pomegranate and gut microbiota: Evidence of health benefits. Crit. Rev. Food Sci. Nutr..

[27] Lim H.J., Kang S.H., Song Y.J., Jeon Y.D., Jin J.S. (2021). Inhibitory Effect of Quercetin on Propionibacterium acnes-induced Skin Inflammation. Int. Immunopharmacol..

[28] Tax G., Urban E., Palotas Z., Puskas R., Konya Z., Biro T., Kemeny L., Szabo K. (2016). Propionic Acid Produced by Propionibacterium acnes Strains Contri-butes to Their Pathogenicity. Acta Derm. Venereol..

[29] Akaza N., Akamatsu H., Kishi M., Mizutani H., Ishii I., Nakata S., Matsunaga K. (2009). Effects of Propionibacterium acnes on various mRNA expression levels in normal human epidermal keratinocytes in vitro. J. Dermatol..

[30] Jarrousse V., Castex-Rizzi N., Khammari A., Charveron M., Dreno B. (2007). Modulation of integrins and filaggrin expression by Propionibacterium acnes extracts on keratinocytes. Arch. Dermatol. Res..

[31] Ng D.C., Shafaee S., Lee D., Bikle D.D. (2000). Requirement of an AP-1 site in the calcium response region of the involucrin promoter. J. Biol. Chem..

[32] Boukamp P., Petrussevska R.T., Breitkreutz D., Hornung J., Markham A., Fusenig N.E. (1988). Normal keratinization in a spontaneously immortalized aneuploid human keratinocyte cell line. J. Cell Biol..

[33] Cerulli A., Napolitano A., Hosek J., Masullo M., Pizza C., Piacente S. (2021). Antioxidant and In Vitro Preliminary Anti-Inflammatory Activity of Castanea sativa (Italian Cultivar “Marrone di Roccadaspide” PGI) Burs, Leaves, and Chestnuts Extracts and Their Metabolite Profiles by LC-ESI/LTQOrbitrap/MS/MS. Antioxidants.

[34] Cerulli A., Masullo M., Mari A., Balato A., Filosa R., Lembo S., Napolitano A., Piacente S. (2018). Phenolics from Castanea sativa leaves and their effects on UVB-induced damage. Nat. Prod. Res..

[35] Sanz M., Cadahia E., Esteruelas E., Munoz A.M., Fernandez de Simon B., Hernandez T., Estrella I. (2010). Phenolic compounds in chestnut (Castanea sativa Mill.) heartwood. Effect of toasting at cooperage. J. Agric. Food Chem..

