# Supplementary material for: Investigation into the Anti-Acne Effects of Castanea sativa Mill Leaf and Its Pure Ellagitannin Castalagin in HaCaT Cells Infected with Cutibacterium acnes
Source: Int J Mol Sci. 2024 Apr 27;25(9):4764. doi: 10.3390/ijms25094764 (PMC11084787; doi:10.3390/ijms25094764)
Supplement: Supplementary file 1 [file ijms-25-04764-s001.zip › ijms-2971240-supplementary.pdf]

Supplementary

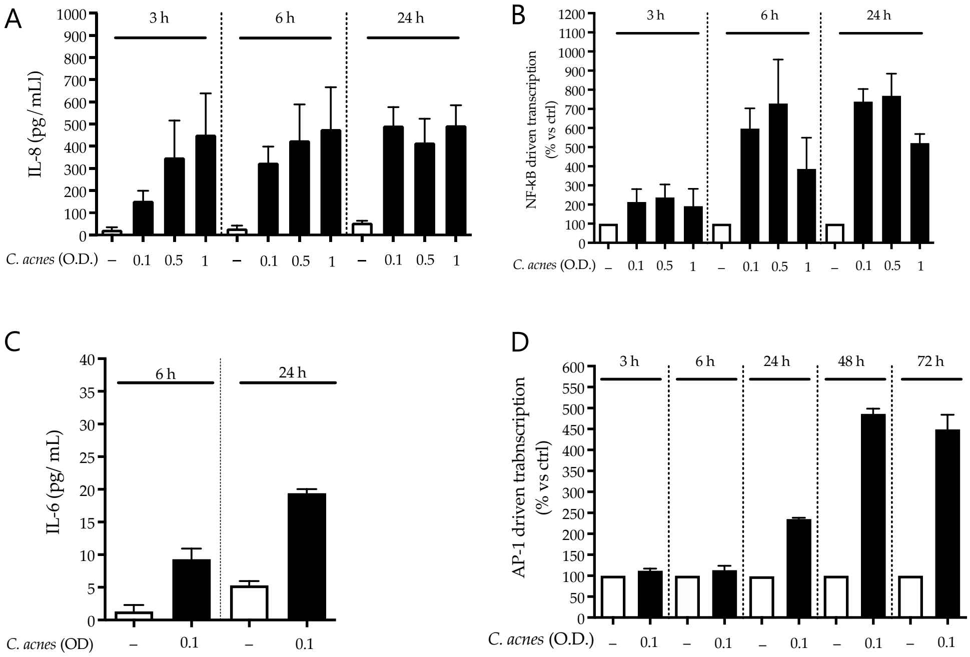

Figure S1. Time-course experiments concerning the inflammatory effect of *C. acnes* on HaCaT cells. Different loads (O.D. $_{600\ nm}$) of *C. acnes* were used to infect HaCaT cells for different time: the release of IL-8 (A) and the NF-κB driven transcription (B) were already increased by O.D.=0.1 and peaked after 24 h. Using the same bacterial load, the increase of IL-6 release (C) and AP-1 driven transcription (D) were measured.

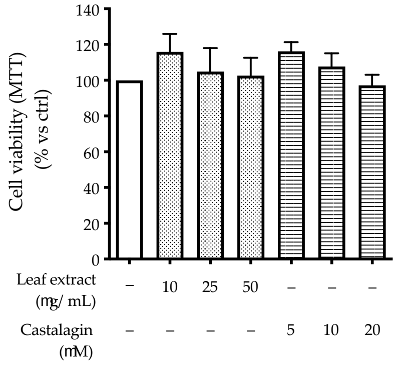

Figure S2. HaCaT cells viability measured by MTT test after treatment with various concentrations of *C. sativa* leaf extract and castalagin for 48 h.
